# Lup-20(29)-en-3β,28-di-yl-nitrooxy acetate affects MCF-7 proliferation through the crosstalk between apoptosis and autophagy in mitochondria

**DOI:** 10.1038/s41419-017-0255-5

**Published:** 2018-02-14

**Authors:** Xiaoning Yan, Lei Yang, Gaili Feng, Zhuli Yu, Minjie Xiao, Weibin Cai, Yanmei Xing, Shasha Bai, Junqia Guo, Zhiyu Wang, Tao Wang, Rong Zhang

**Affiliations:** 1 Institute of Clinical Pharmacology, Guangzhou University of Chinese Medicine, 510405 Guangzhou, China; 2 The Collaborative Innovation Center of Comprehensive Development and Utilization of Shanxi Chinese Mdicine, Shanxi University of Chinese Medicine, 030600 Jinzhong, China; 3 School of Chinese Meteria Medica, Guangzhou University of Chinese Medicine, 510006 Guangzhou, China; 4 Guangdong Provincial Hospital of Chinese Medicine, Guangzhou University of Chinese Medicine, 510006 Guangzhou, China

## Abstract

Betulin (BT), a pentacyclic lupine-type triterpenoid natural product, possesses antitumor activity in various types of cancers. However, its clinical development was discouraged due to its low biological activities and poor solubility. We prepared lup-20(29)-en-3β,28-di-yl-nitrooxy acetate (NBT), a derivative of BT, that was chemically modified at position 3 of ring A and C-28 by introducing a NO-releasing moiety. This study mainly explored the mechanism of NBT in treating breast cancer through the crosstalk between apoptosis and autophagy in mitochondria. NBT possessed a potent antiproliferative activity in MCF-7 cells both in vitro and in vivo. Mechanically, NBT affected cell death through the mitochondrial apoptosis pathway and autophagy. NBT induced cell cycle arrest in the G_0_/G_1_ phase by decreasing the expression of cyclin D1. It also induced mitochondrial apoptosis by increasing the expression of Bax, caspase-9, and poly(ADP-ribose) polymerase and mitochondrial membrane potential loss and leaks of cytochrome c (Cyt C) from mitochondria in MCF-7 cells and decreasing the expression of mitochondrial Bcl-2. We further demonstrated whether chloroquine (CQ), which inhibits the degradation of autophagosome induced by NBT, affects the proliferation of MCF-7 cells compared with NBT. The experiments inferred that the combination of NBT and CQ significantly promoted MCF-7 cell mitochondria to divide and Cyt C to be released from mitochondria to the cytoplasm, resulting in an increased apoptosis rate. The in vivo experiments showed that NBT inhibited the growth of MCF-7 tumor via the apoptosis pathway, and its effect was similar to 5-fluorouracil.

## Introduction

**Fig. 1:**
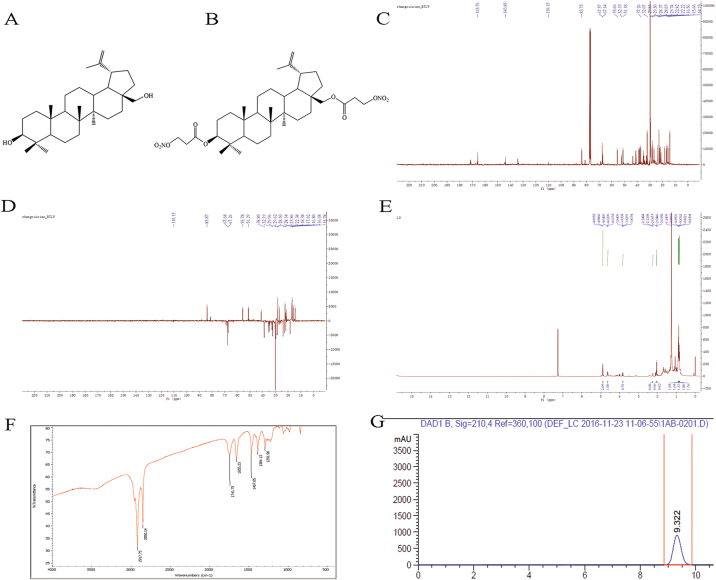
Structures of BT and NBT. **a** Chemical structure of BT. **b** Chemical structure of NBT. **c** ^13^C NMR chromatogram of NBT. **d** DEPT 135 chromatogram of NBT. **e** ^1^H NMR chromatogram of NBT. **f** IR chromatogram of NBT. **g** HPLC chromatogram

Betulin (BT) (Fig. 1a) is a naturally occurring pentacyclic lupine-type triterpenoid from birch bark extract with potential hepatoprotective^1^, anti-inflammatory^2^, anti-HIV^3^, antiproliferative^4^, and anticancer^5^ properties. In addition, the antitumor activity of BT has been observed in a broad range of cancer cell lines, and it has demonstrated potent inhibition of proliferation in solid tumors by activating the mitochondrial apoptosis pathway characterized by the cleavage of caspases and poly(ADP-ribose) polymerase (PARP), attenuation of Bcl-2, mitochondrial depolarization, and chromatin condensation^6–8^. Despite reports of good efficacy and safety of BT in tumor therapy, its clinical application is discouraged because of its low bioavailability and poor solubility. We focused on the modification of BT at the C-3 and/or C-28 positions as modifications at these positions have been reported to improve its antitumor and antimicrobial activities and hydrosolubility^9^. Nitric oxide (NO), an important endogenously produced cell signaling and target molecule involved in many physiological and pathological reactions, plays a significant anticancer role via the toxicity of macrophage to tumor cells, inhibition of angiogenesis and metastasis, proliferation inhibition, and apoptosis of tumor cells in various types of cancer cells^10–12^. We introduced a NO-releasing moiety into BT by targeting position 3 of ring A and C-28 to synthesize a library of different NO-releasing derivatives of BT by considering the evidence that NO at high concentrations exhibits tumoricidal activity, whereas at low concentrations it stimulates tumor proliferation^13^ and mediates apoptosis via intrinsic apoptotic signaling by down-regulating Bcl-2 expression^14^. Among the various derivatives, lup-20(29)-en-3β,28-di-yl-nitrooxy acetate (NBT) (Fig. 1b) was the most effective in inhibiting cancer cells, especially in HepG 2 and MCF-7 cells, as evidenced in our previous study ^15^.

Apoptosis and autophagy participate in cellular degradation pathways for maintaining cellular homeostasis and are involved in the protection of organisms from cancer^16–18^. Apoptosis, a major way of killing cancer cells by anticancer agents, includes two kinds of pathways: caspase-dependent and caspase-independent. The caspase-dependent pathway mostly occurs through extrinsic or intrinsic pathways^19^. Mitochondria are of great significance in intrinsic apoptosis. Autophagy is a conserved process that is involved in turning over organelles, protein degradation, and differentiation^20^. It begins with the trimer formation of beclin 1, PI3KC3 (Vps34), and Atg 14, with beclin 1 constantly increasing autophagy-related proteins. Light chain 3-II (LC3-II) plays an important role in the elongation of the double membrane until formation of the autolysosome, through the fusion of mature autophagosome and lysosome^21^. Atg5 is required for LC3 lipidation in autophagy and switches autophagy to apoptosis^22^. p62, a multifunctional protein, combines with ubiquitinated protein and binds to LC3 II proteins to form a complex that is eventually degraded by enzymes in the lysosome when autophagy occurs^23,24^. Hence, it is constantly consumed with increasing levels of autophagy. Therefore, Atg-5, beclin-1, LC 3-II, and p62 are major indicators in the development of autophagy ^25,26^.

Autophagy can evidently reduce the potency of therapeutic agents for cancers via increasing cellular survival in stress conditions^27,28^. In this study, we sought to evaluate the effect of NBT on inhibiting the proliferation of MCF-7 cells in vitro and in vivo and attempted to elucidate its anticancer mechanisms in terms of apoptosis, autophagy, and the relationship between apoptosis and autophagy.

## Results

### NBT structure

NMR (Fig. 1c–e) and IR (Fig. 1f) were used to identify the structure of NBT. NBT was analyzed by using HPLC (Fig. 1g) and found to be 99.9% pure. ^13^C NMR (CDCl_3_, 100 MHz): *δ* 38.5 (C-1), 23.6 (C-2), 83.6 (C-3), 40.8 (C-4), 55.5 (C-5), 18.1 (C-6), 34.6 (C-7), 43.3 (C-8), 51.0 (C-9), 37.1 (C-10), 21.5 (C-11), 22.6 (C-12), 37.6 (C-13), 51.0 (C-14), 28.2 (C-15), 31.9 (C-16), 37.9 (C-17), 52.1 (C-18), 48.8 (C-19), 144.1 (C-20), 29.3(C-21), 34.9 (C-22), 27.9 (C-23), 16.7 (C-24), 16.5 (C-25), 15.5 (C-26), 14.1 (C-27), 67.0 (C-28), 109.6 (C-29), 21.0 (C-30), 165.6 (C-31, C-31’), 67.4 (C-32, C-32’); ^1^H NMR (CDCl_3_, 400 MHz): *δ* 0.83, 0.85, 0.86, 0.87, 0.89, 1.04 (s, 18 H, 6 × CH_3_), 2.25 (m, 1 H, H-19), 4.61 (d, 1 H, *J* = 7.5 Hz, H-29b), 4.64 (d, 1 H, *J* = 7.5 Hz, H-29a), 3.83 (m, 2 H), 4.89, 4.88 (s, 2 × CH_2_ONO_2_); IR (KBr) cm^−1^: 2918, 2850, 1742, 1655, 1467, 1384, 1292.

### NBT inhibits the proliferation of cancer cells

**Fig. 2:**
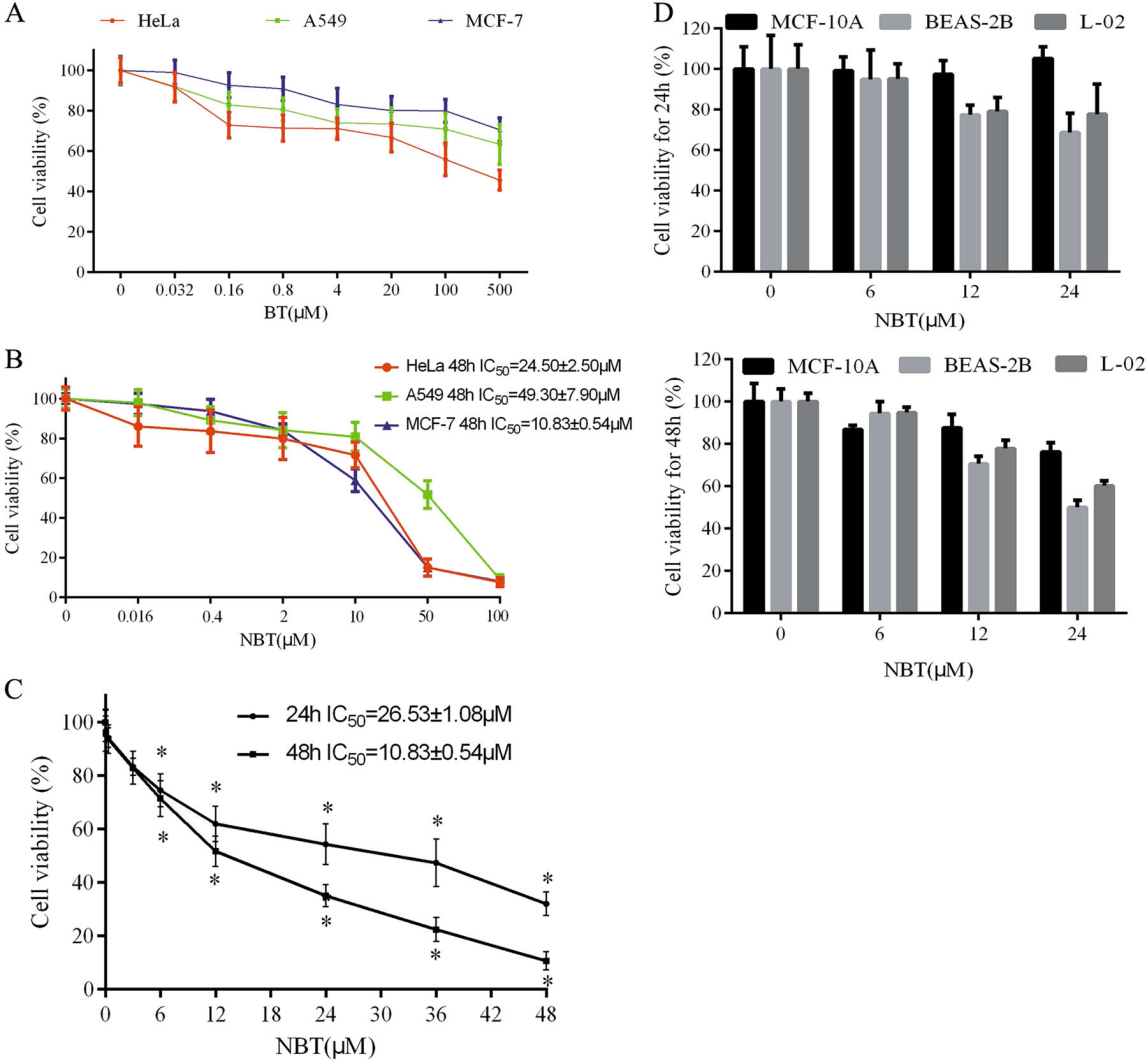
NBT and BT induced cell death in HeLa, A549, and MCF-7 cell lines. **a** Effects of BT on the proliferation of MCF-7, HeLa, and A549 cells. Cells (5 × 10^3^) were seeded in a 96-well plate for 24 h and then treated with increased concentrations of BT for 48 h. The cell viability was evaluated by using CCK-8 assay. **b** MCF-7, HeLa, and A549 cells (5 × 10^3^) were seeded in a 96-well plate for 24 h and were treated with known concentrations of NBT for 48 h. **c** MCF-7 cells (5 × 10^3^) were seeded in a 96-well plate for 24 h and then were treated with known concentrations of NBT for 24 or 48 h. The cell viability was evaluated by using CCK-8 assay. **d** MCF10A, BEAS-2B, and L-02 cells (5 × 10^3^) were seeded in a 96-well plate for 24 h and were treated with 6, 12, and 24 μM concentrations of NBT for 24 or 48 h. The cell viability was evaluated by using CCK-8 assay. Data represent mean ± SD (*n* = 3) and are representative of triplicate experiments (^*^P < 0.05, vs. control group)

As seen in Fig. 2a, b, the IC_50_ values of NBT for HeLa, A549, and MCF-7 were 24.50 ± 2.50, 49.30 ± 7.90, and 10.83 ± 0.54 μM, respectively, for 48 h. The IC_50_ value of BT for HeLa was 288.21 ± 12.30 μM, and the A549 and MCF-7 treated with BT at 500 μM exhibited ≤40% inhibition (Fig. 2a). Furthermore, NBT inhibited MCF-7 cell proliferation in a time-dependent and dose-dependent manner (Fig. 2c). In addition, the possible toxicity of NBT was detected in human normal cells (MCF10A, BEAS-2B, and L-02). At 6, 12, and 24 μM for 24 h, NBT did not show any toxic effects on the abovementioned cell lines. However, BEAS-2B cells treated with NBT at 24 μM for 48 h exhibited 50% inhibition (Fig. 2d).

### NBT activates the G_0_/G_1_ checkpoint in MCF-7 cells

**Fig. 3:**
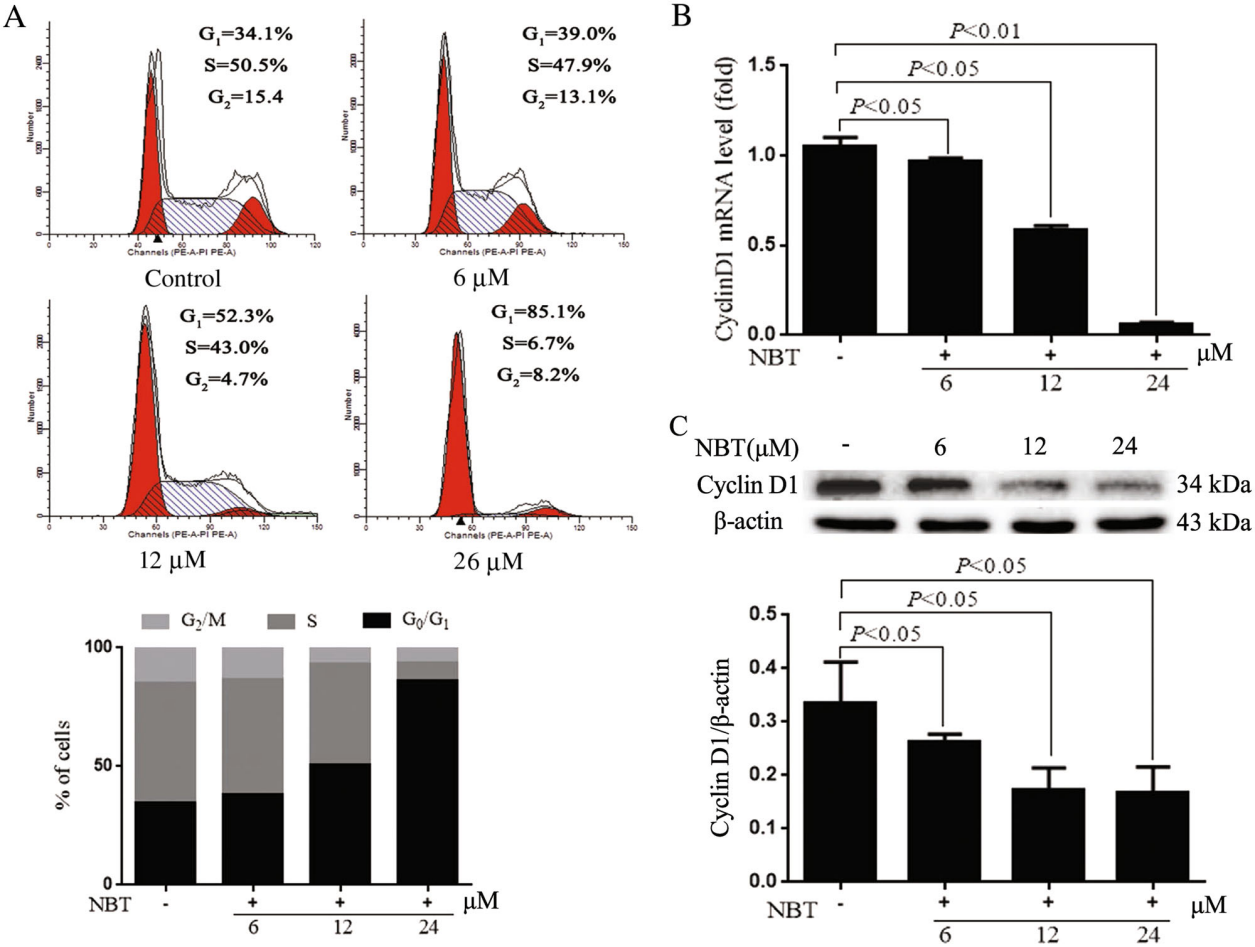
NBT affected the cell cycle in MCF-7 cells. **a** Flow cytometry assay for the cell cycles of MCF-7 cells treated with or without 6, 12, and 24 μM NBT for 24 h. **b** Quantitative real-time PCR assay for cyclin D1 in MCF-7 cells treated with or without 6, 12, and 24 μM NBT for 24 h. GADPH was the housekeeping control. **c** Western blot and analysis of cyclin D1 after 24 h of NBT treatment (*n* = 3 in triplicate, ANOVA)

Cell cycle was tested to determine whether NBT changes the cell cycle in MCF-7 cells. At 24 μM of NBT for 24 h, 41.0% accumulation was found in the G_0_/G_1_ phase compared with untreated controls, suggesting that NBT can induce cell arrest in the G_0_/G_1_ phase. A dose-dependent cell cycle arrest in the G_1_ phase was observed (Fig. 3a). Furthermore, the mRNA and protein levels of cyclin D1 were analyzed in comparison with the control. Both cyclin D1 mRNA and protein were significantly decreased in NBT-treated groups in a dose-dependent manner (Fig. 3b, c).

### Mitochondrial pathway plays a key role in apoptotic cell death induced by NBT

**Fig. 4:**
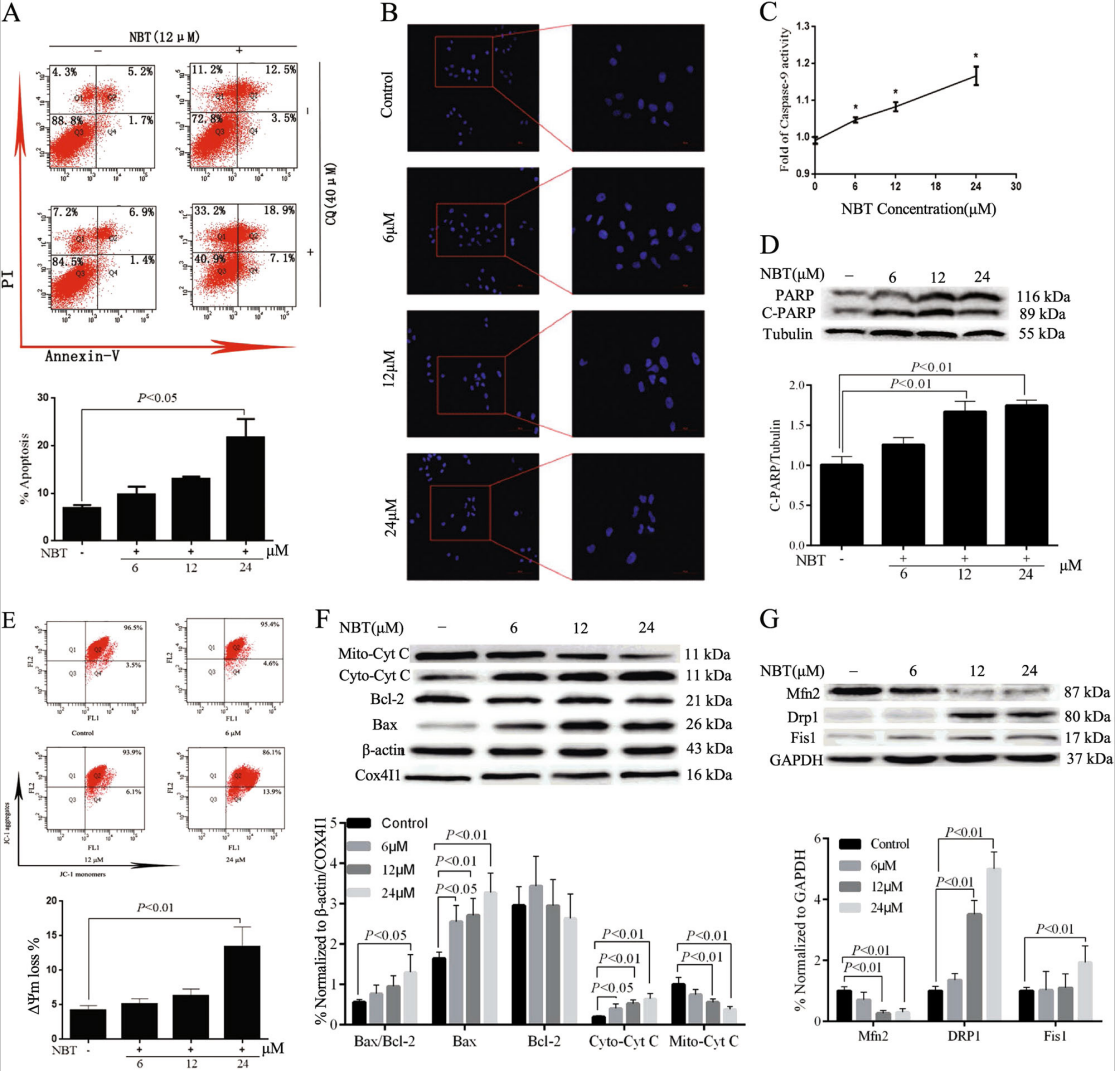
NBT induced MCF-7 cell apoptosis through the mitochondrial pathway. **a** MCF-7 cells were treated with various concentrations of NBT for 24 h, and apoptosis was examined by using annexin V with PI staining with flow cytometry. **b** Hoechst 33258 staining assay showed that NBT at doses of 6, 12, and 24 μM induced chromatin shrinking of MCF-7 cells (×200, scale bars: 100 μm; ×400, scale bars: 50  μm). **c** Treatment of 6, 12, and 24 μM NBT influenced caspase-9 activity in MCF-7 cells. **d** Total cellular extract of MCF-7 cells treated with 6, 12, and 24 μM NBT was prepared and subjected to Western blot by using an antibody against PARP. **e** JC-1 staining assay determined the ΔΨm in mitochondria of MCF-7 cells treated with 6, 12, and 24 μM NBT. **f** Total cellular extract, mitochondrial fraction, and cytosol fractions of MCF-7 cells treated with 6, 12, and 24 μM NBT were prepared and subjected to Western blot by using antibodies against Bcl-2, Bax, and Cyt C (mitochondrial and cytosol fraction). **g** Total cellular extract of MCF-7 cells treated with 6, 12, and 24 μM NBT was prepared and subjected to Western blot by using antibodies against DRP1, Fis1, and Mfn2. Data represent mean ± SD of three independent experiments (^*^P < 0.05, vs. control group)

We also attempted to determine whether the cytotoxic effect of NBT is associated with apoptosis. The results indicated that the apoptotic rates of MCF-7 cells treated with NBT were higher than those of the control in a concentration-dependent manner. The apoptotic cell populations increased up to 24.6% in MCF-7 cells at 24 μM of NBT (Fig. 4a). Furthermore, Hoechst 33258 assay results showed brighter nuclei staining, condensation of nuclear chromatin, and karyorrhexis, which indicated more cell apoptosis in NBT-treated groups than in control (Fig. 4b).

To further determine the apoptotic response to NBT treatment, caspase-9 activity and PARP levels were assessed after 24 h of NBT treatment at 6, 12, and 24 μM. Caspase-9 activity was dose dependently increased in the NBT treated groups compared with the control (Fig. 4c). Meanwhile, the cleavage of PARP expression showed similar change (Fig. 4d). These results demonstrated that NBT induced cell apoptosis in MCF-7 cells in a dose-dependent manner.

The ΔΨm loss of MCF-7 cells increased to 4.6%, 6.1%, and 13.9% with NBT treatment at 6, 12, and 24 μM, compared with 3.5% in the control (Fig. 4e). The loss of ΔΨm directly reflected the effect of NBT on mitochondrial function, which could activate the caspase family and then cause cell apoptosis in MCF-7 cells.

Antiapoptotic Bcl-2 and proapoptotic Bax proteins mainly regulate ΔΨm accompanied by cytochrome c (Cyt C) released from mitochondria^29^. These proteins were examined to investigate the underlying mechanism of the proapoptotic effect of NBT on MCF-7 cells. Compared with the control, the Bax/Bcl-2 ratio increased progressively after NBT treatment, especially at 24 μM (nearly 2.22-fold). Bax was up-regulated and Bcl-2 was down-regulated in MCF-7 cells treated with NBT (Fig. 4f). In addition, NBT significantly increased the cytoplasmic Cyt C and decreased the mitochondrial Cyt C (Fig. 4f).

Mitochondrial fission plays a critical role in mitochondrial apoptosis, which leads to DRP1- and Fis1-induced mitochondrial fragmentation and decreased levels of fusion proteins Mfn1 and Mfn2^30,31^. Western blot results showed that NBT increased DRP1 and Fis1 but decreased Mfn2 in a dose-dependent manner (Fig. 4g).

### NBT promotes the autophagic flux

**Fig. 5:**
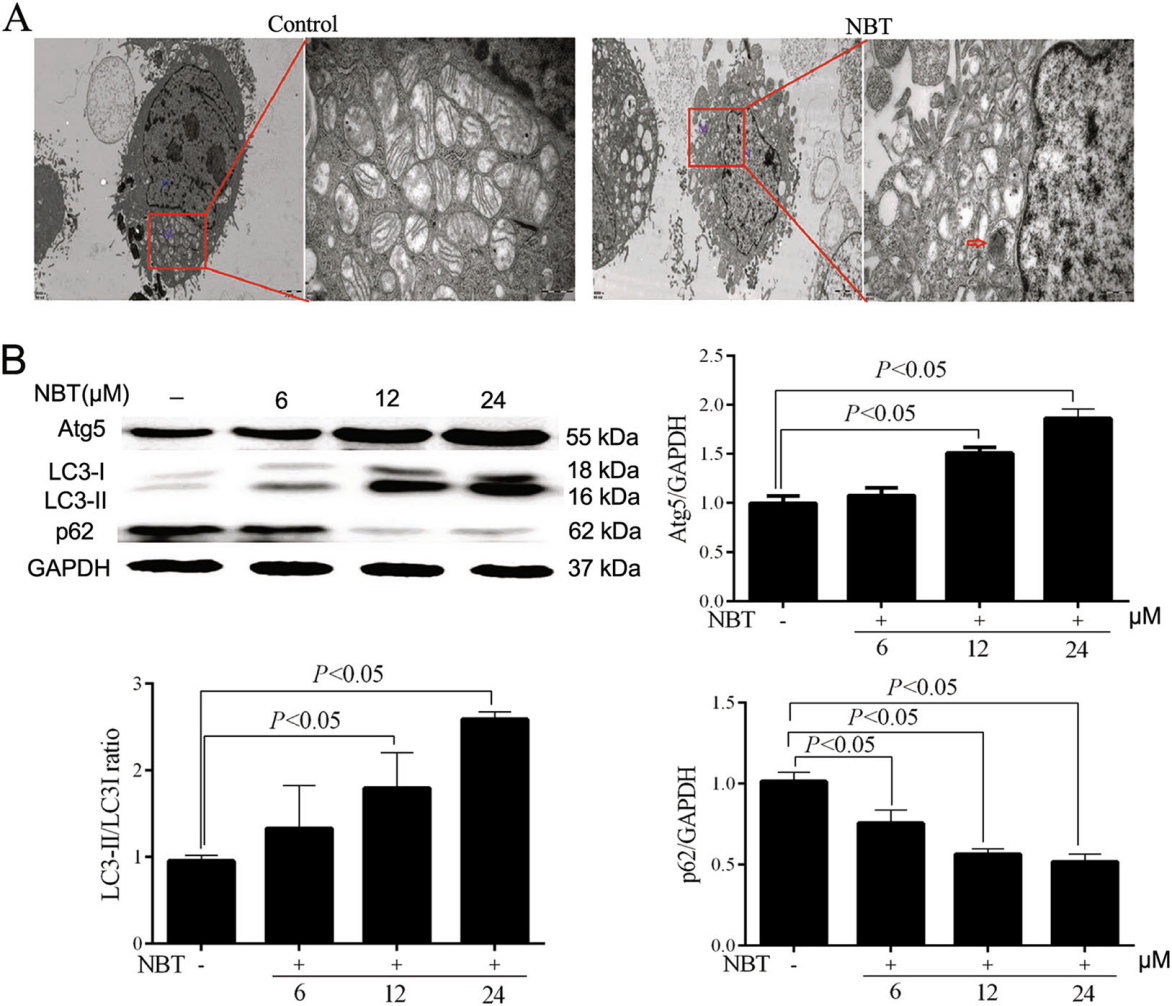

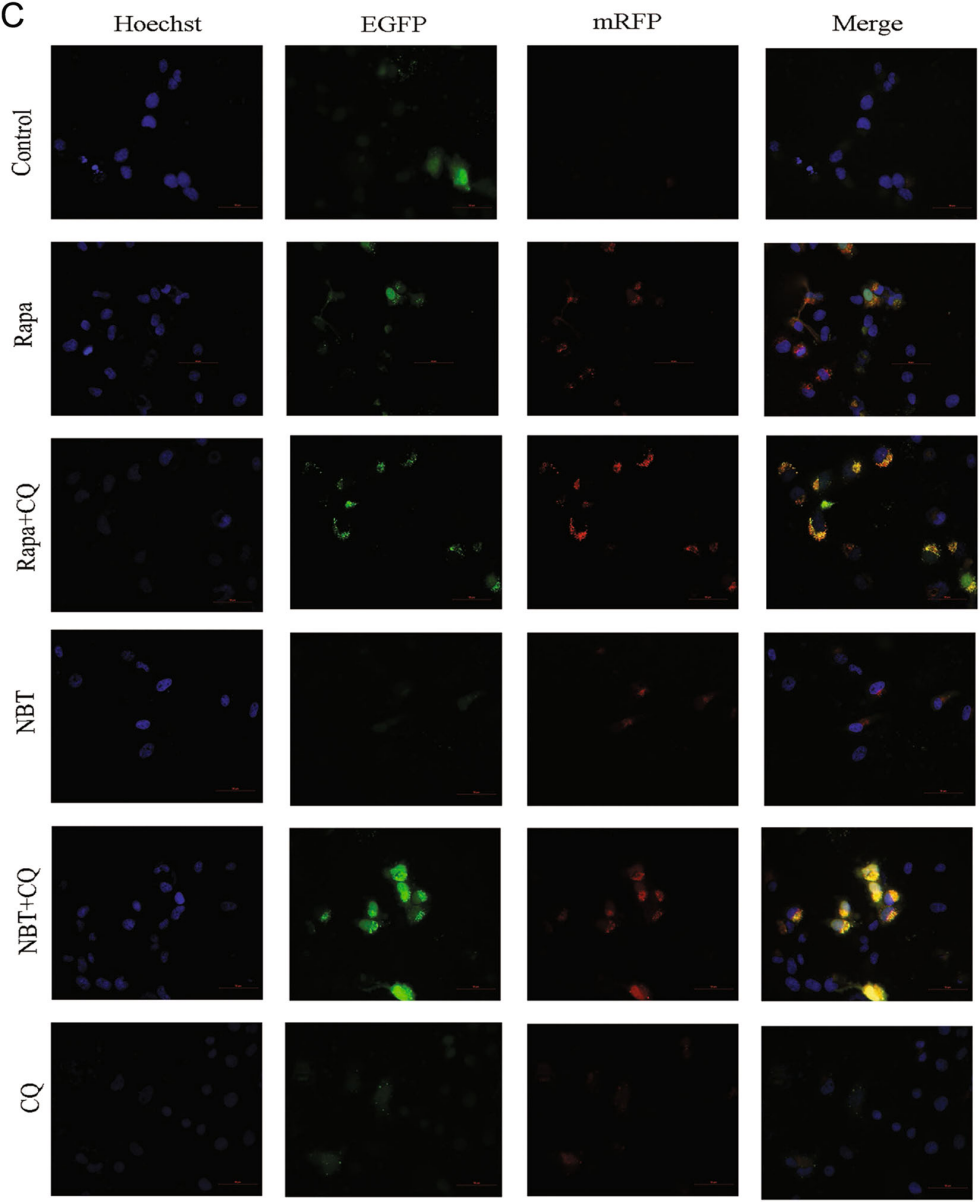
NBT induced autophagic flux in MCF-7 cells. **a** Representative TEM images of MCF-7 cells treated with NBT (12 μM). N nucleus; M mitochondria; red arrows indicate autophagic vacuoles (scale bars: 2 μm, 500 nm). **b** Total cellular extract of MCF-7 cells treated with 6, 12, and 24 μM NBT was prepared and subjected to Western blot by using antibodies against Atg5, LC3B, and p62. Data represent mean ± SD of three independent experiments. **c** Cells were transfected with a tandem reporter construct (tfLC3) for 48 h and were exposed to Rapa (0.25 μM), NBT (12 μM), and/or CQ (40 μM) for 24 h. The cells were then stained with Hoechst 33258 fluorescent dye. The puncta were examined by using a fluorescence microscope. Scale bars: 50 μm

NBT treatment induced the formation of a double membrane and consequently degraded autophagic vacuoles. In addition, a few vesicles appeared in the ectoplasm. Thus, transmission electron microscope (TEM) images indicated that NBT induced autophagy and apoptotic changes in MCF-7 cells (Fig. 5a). Three key indicators for autophagy, i.e., Atg5, LC3 I/II, and p62, were examined to investigate whether NBT induces autophagy in MCF-7 cells. Western blot results showed that the Atg5 level and the ratio of LC3 II/LC3 I were remarkably increased in MCF-7 cells treated with NBT for 24 h (Fig. 5b). Moreover, the potentiation of autophagy induced by NBT treatment was further evidenced by the reduction of p62 (Fig. 5b). These results implied that NBT induced the autophagic flux.

To assess the autophagic flux, MCF-7 cells were transiently transfected with the mRFP-GFP-LC3 vector. mRFP is stable under acidic conditions, whereas GFP is not present in the acid environment of autolysosome. Rapamycin (Rapa), which induces autophagy by inhibiting the mTOR pathway, was chosen as the positive agent. Chloroquine (CQ), which inhibits the fusion of autophagosome and lysosome, was used for autophagic flux detection. Rapa treatment induced yellow and red puncta, which respectively denote autophagosomes and autolysosomes (Fig. 5c). MCF-7 cells exposed to both Rapa and CQ produced autophagosomes (yellow) but only few autolysosomes (red) (Fig. 5c). The puncta induced by Rapa were mimicked by NBT treatment (Fig. 5c). Furthermore, we observed brighter nuclei staining and condensation of nuclear chromatin stained with Hoechst 33258 fluorescent dye in the transfected MCF-7 cells with a plasmid harboring a tandem fluorescent mRFP-GFP-LC3 (Fig. 5c). The data suggested that NBT not only induced apoptosis but also elicited the complete autophagic process in MCF-7 cells.

### NBT combined with CQ induces mitochondrial apoptosis via excessive accumulation of autophagosomes/mitophagosomes

**Fig. 6:**
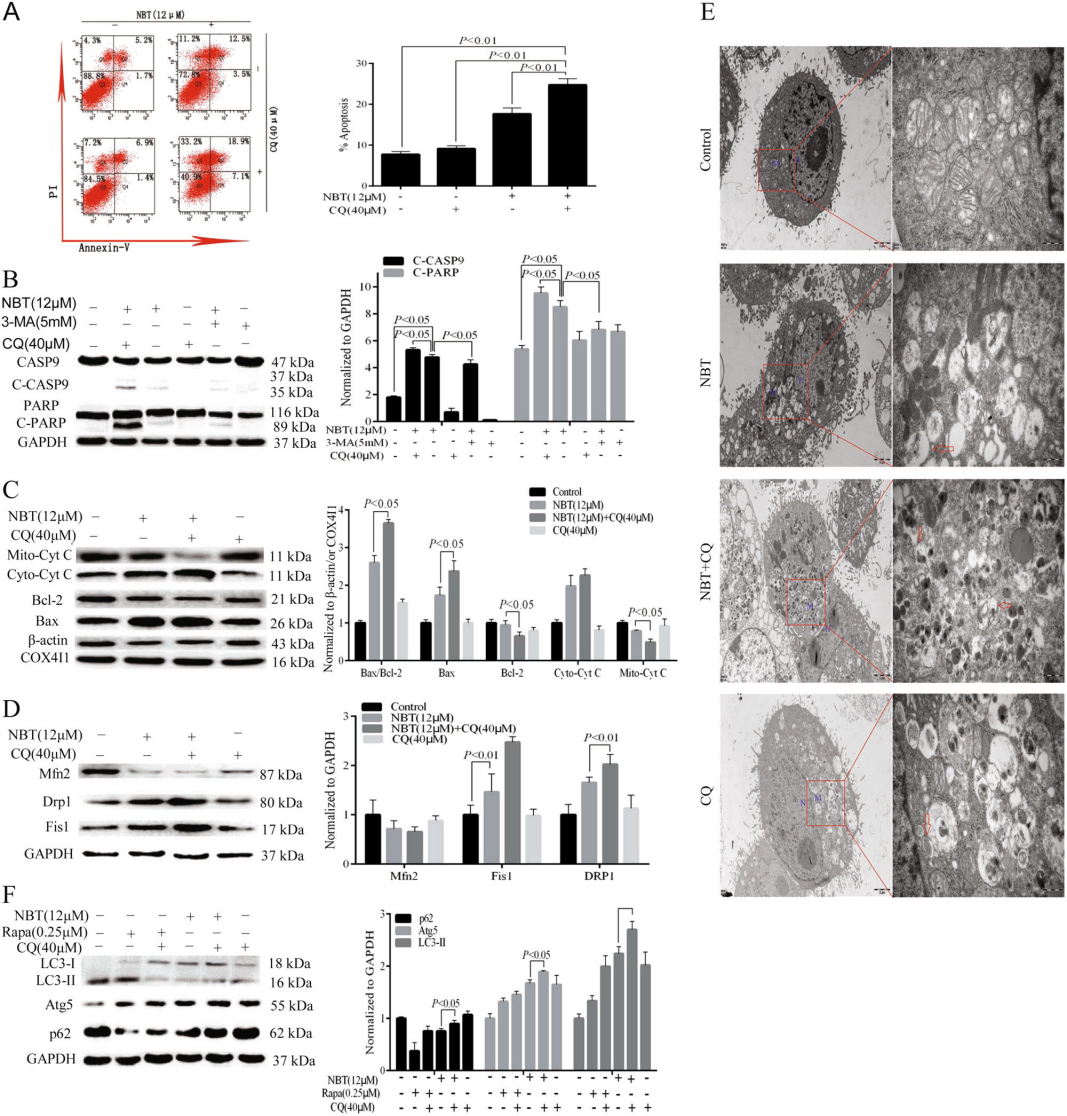
Inhibiting autophagy enhanced NBT-induced apoptosis. **a** MCF-7 cells were cotreated with 12 μM NBT and 40 μM CQ for 24 h compared with control or NBT and CQ treatment alone, and apoptosis was examined by using annexin V with PI staining with flow cytometry. **b** MCF-7 cells were coexposed to NBT (12 μM) and CQ (40 μM) in the presence or absence of 3-MA (5 mM) for 24 h. Total cellular extract of MCF-7 cells was prepared and subjected to Western blot by using antibodies against CASP9 and PARP. **c** Total cellular extract, mitochondrial fraction, and cytosol fractions of MCF-7 cells was prepared and subjected to Western blot by using antibodies against Bcl-2, Bax, and Cyt C (mitochondrial fraction and cytosol fraction). **d** Total cellular extract of MCF-7 cells was prepared and subjected to Western blot by using antibodies against DRP1, Fis1, and Mfn2. **e** Representative TEM images of MCF-7 cells. MCF-7 cells were exposed to NBT (12 μM) in the presence or absence of CQ (40 μM) for 24 h. N nucleus; M mitochondria; red arrows indicate autophagic vacuoles (scale bars: 2 μm, 500 nm). **f** Total cellular extract of MCF-7 cells cotreated with 40 μM CQ and 12 μM NBT or 0.25 μM Rapa for 24 h compared with control or NBT, Rapa, and CQ treatment alone was prepared and subjected to Western blot by using antibodies against Atg5, LC3B, and p62. Data represent mean ± SD of three independent experiments

The cotreatment of NBT and CQ for 24 h in MCF-7 cells resulted in a marked increase in apoptosis according to the annexin V/propidium iodide (PI) staining test (Fig. 6a). A 10% increase was observed in the apoptotic population with cotreatment of NBT and CQ (26.0%) compared with NBT alone (16.0%). Meanwhile, the cotreatment of NBT and CQ resulted in increased caspase-9 and PARP activation (Fig. 6b). However, 3-methyladenine (3-MA), which inhibits autophagosome formation, was observed to inhibit NBT-induced caspase-9 and PARP activation in MCF-7 cells (Fig. 6b). The combination of NBT and CQ resulted in a significant increase in Bax/Bcl-2 and Cyt C (Fig. 6c). In addition, the effects of the combination of NBT and CQ on mitochondrial dynamics were examined. We found that the combination of NBT and CQ enhanced DRP1 and Fis1 levels but decreased Mfn2 levels compared with NBT or CQ treatment alone (Fig. 6d). TEM results showed that the combination of NBT and CQ increased the amounts of damaged swollen mitochondria, and more autophagic/mitophagic vacuoles were observed (Fig. 6e).

Furthermore, Rapa increased Atg5 and LC3B-II levels, which were further increased by CQ (Fig. 6f). Rapa decreased the level of p62 protein, which was enhanced by CQ (Fig. 6f). In addition, the combination of NBT and CQ increased the levels of Atg5, LC3B-II, and p62, which resulted in an excessive accumulation of autophagosomes/mitophagosomes (Fig. 6f). Collectively, these results indicated that inhibiting autophagy can enhance NBT-induced mitochondrial fission, resulting in more apoptotic MCF-7 cells through the accumulation of autophagosomes/mitophagosomes.

### NBT inhibits the growth of MCF-7-derived xenograft in nude mice

**Fig. 7:**
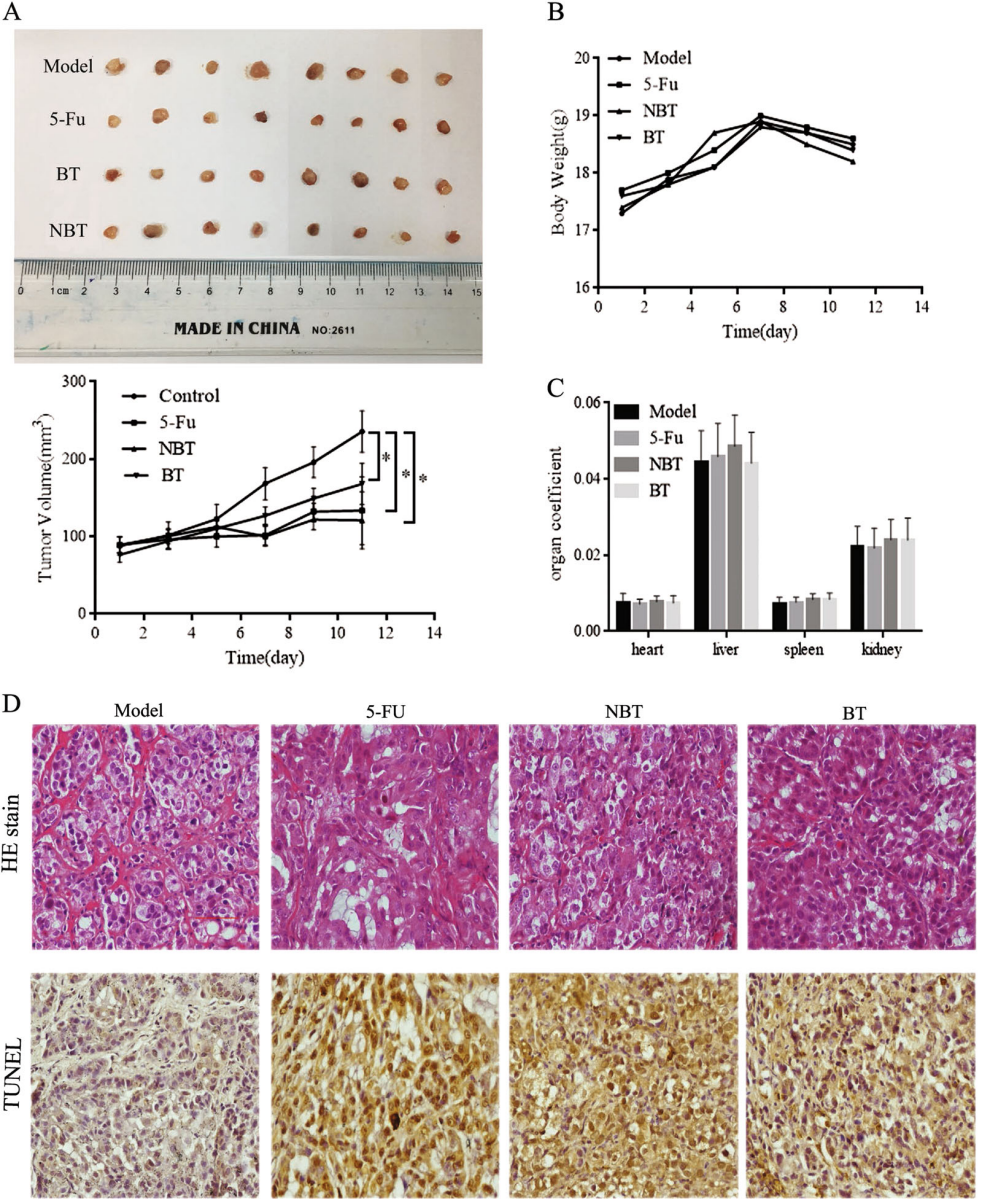
NBT inhibited the growth of MCF-7 solid tumor in a MCF-7 mouse xenograft model. **a** Average tumor volume of MCF-7 xenografts in vehicle model mice and mice treated with NBT (100 mg/kg), BT (100 mg/kg), or 5-FU (22 mg/kg) for 11 days. ^*^P < 0.05, vs. control group. **b** Body weight of mice during 11 days of exposure. Statistical analysis showed no significant differences in body weight changes. **c** Coefficient of the heart, liver, spleen, and kidney to the body weight of mice after 11 days of exposure. Statistical analysis showed no significant differences. **d** H&E staining and TUNEL assay for examining histological morphology and apoptosis. Representative images are shown at ×400 magnification. Scale bar: 100 μm. Data represent mean ± SD of three independent experiments

The antitumor activities of NBT and BT in vivo were evaluated. After administering NBT at 100 mg/kg i.p. and BT at 100 mg/kg i.p. for 11 days, NBT or 5-fluorouracil (5-FU) led to a significant reduction in tumor growth compared with the control, whereas BT treatment caused minimal reduction in tumor growth (Fig. 7a). No significant changes were observed in body weight, organ coefficient, and biochemical parameters (Fig. 7b, c, Table 1).

**Table 1:** Mean values of various biochemical parameters measured from serum of female mice treated with vehicle, BT and NBT ($\bar X \pm S$, *N* = 8)

Parameters	ALT/GPT (IU/L)	AST/GOT (IU/L)	BUN (mmol/L)	CRE (μmol/L)	UA (μmol/L)
Model	82.35 ± 17.42	153.52 ± 27.67	8.12 ± 1.24	36.89 ± 8.78	96.16 ± 6.26
5-FU	95.48 ± 12.86	245.71 ± 47.15	9.26 ± 2.23	32.95 ± 6.29	84.52 ± 6.63
NBT	126.47 ± 8.41	162.85 ± 32.48	8.32 ± 2.95	37.87 ± 8.54	126.74 ± 9.98
BT	106.33 ± 24.27	183.42 ± 39.98	10.13 ± 3.04	45.10 ± 6.43	130.18 ± 25.22

^*^P < 0.05, by ANOVA

HE staining results showed that fibrosis, necrosis, and inflammatory cell infiltration were observed in tumors treated with NBT or BT (Fig. 7d, top panels). TUNEL results showed that similar to 5-FU, NBT treatment caused more TUNEL-positive cells (brown color) than BT (Fig. 7d, bottom panels).

## Discussion

Breast cancer causes the most cancer-related deaths among females^32^. According to the World Health Organization, more than 1.7 million women were diagnosed with breast cancer all over the world in 2012^33^. Although chemotherapeutics are widely used for breast cancer treatment, certain shortcomings, such as off-target effects, remain major therapeutic problems^34^. Natural and chemically modified natural products exhibit inhibitions at different stages of the cancer growth process. Hence, there is an urgency to develop more efficient molecules that selectively inhibit cancer cell growth. In this study, we found that NBT, a novel semisynthetic derivative of BT, possesses a potent antiproliferative activity in MCF-7 cells by inducing cell death. Mechanically, NBT induces apoptosis and elicits autophagy. Moreover, for the first time, we have demonstrated that inhibition at late-stage autophagy can enhance NBT-induced apoptosis. However, we found that NBT showed no toxic effects on normal cells except for BEAS-2B cells at 24 μM concentration. When MCF-7 cells were treated at a higher concentration (24 μM) of NBT for a long time (48 h), BEAS-2B cells were found to be sensitive to NBT, exhibiting 50% inhibition. Therefore, 24 h was selected as the optimal time point to perform further experiments. The results meant a possible side effect of NBT on lung function.

Different molecular mechanisms have been implicated in the anticancer activities of chemotherapeutics. Novel molecules selectively inhibit the proliferation of cancer cells via the PI3K/AKT signaling pathway, which induces cell cycle progression and regulates cell survival and apoptosis^35,36^. In this study, we observed that NBT induced cell cycle arrest in the G_0_/G_1_ phase by down-regulating the gene and protein expression levels of cyclin D1 in MCF-7 cells. In addition, NBT augmented a dose-dependent apoptosis induction. Mitochondria play a crucial role in the caspase-dependent apoptosis, which has been validated by evaluating the expression of Bcl-2 family proteins^37^. The expression of Bcl-2 is up-regulated in breast cancer^38^, and the high expression of Bax initiates apoptosis^39^. In the present study, NBT enhanced the Bax/Bcl-2 ratio, increased the loss of ΔΨm, and increased Cyt C release from mitochondria to cytosol. These results confirmed the involvement of mitochondria in NBT-induced apoptosis in MCF-7 cells. Moreover, NBT increased the activity of caspase-9 and cleaved-PARP, suggesting that the NBT-induced cell cycle arrest in the G_0_/G_1_ phase preceded apoptosis. The loss of ΔΨm via the modulation of Bcl-2 family protein expression and Cyt C resulted in the disruption of mitochondrial function. Furthermore, the activation of caspase-9 suggested that a mitochondrial-dependent factor was responsible for its apoptotic potential. Subsequently, the activation of the caspase cascade led to apoptosis after formatting the cleaved products of PARP in MCF-7 cells. These results were consistent with the anticancer mechanism of BT. DRP1 catalyzes mediated mitochondrial fission and is recruited to mitochondria^40^. Mfn1 and Mfn2, which are tethered to the outer mitochondrial membrane to function, drive mitochondrial fusion^41,42^. Our results suggested that mitochondrial dynamic proteins participated in NBT-induced apoptosis in MCF-7 cells. The NBT-induced mitochondrial fission led to the loss of ΔΨm, increased Cyt C in the cytoplasm, activation of caspase-9, and degradation of PARP, which preceded apoptosis.

Autophagy, an adaptation response to stress, regulates the turnover and recycling of organelles in the cytoplasm^43^. Hypoxia and limited nutrients have been reported to activate autophagy, which may help solid tumor cell survival^44^. In addition, autophagy promotes the survival of parts of tumor cells to defend themselves against cancer treatment, and eventually, they become blocks for cancer treatment^45,46^. However, autophagy also has been shown to induce cell death because of excessive stress. NBT treatment resulted in the accumulation of Atg5 and LC3-II levels and decreased the expression of p62 in MCF-7 cells, indicating that it induced autophagy in MCF-7 cells. In addition, our findings clearly indicated that NBT treatment induced the autophagic flux in MCF-7 cells, where the numbers of autophagosome and LC3 puncta accumulated. Previous studies have shown that autophagy induced by inhibiting p53 led to cell cycle arrest in the G_1_ phase^47,48^. We inferred that NBT might induce cell cycle arrest in the G_1_ phase by inhibiting the expression level of p53 in the MCF-7 cells.

The link between apoptosis and autophagy is complicated with the prosurvival or anticancer effect of autophagy. The studies in clinical trials seem to support that the efficacy of anticancer drugs is enhanced by inhibiting autophagy^49,50^. Moreover, a study reported that blocking the autophagic flux in breast cancer MCF-7 cells can induce cell death^51^. In the present study, NBT not only induced apoptosis but also elicited the complete autophagic process in MCF-7 cells. The results indicated that disturbing the autophagic flux enhanced the apoptosis induced by NBT in MCF-7 cells. 3-MA affects autophagy at an early stage via inhibiting its formation, whereas CQ inhibits the fusion of autophagy with lysosome at a later stage^52^. Currently, the clinical drugs for inhibiting autophagy only include CQ and hydroxychloroquine^53^. CQ and 3-MA were chosen as autophagic inhibitors to test the effect of inhibiting autophagy on NBT-mediated apoptosis in MCF-7 cells. In our experiments, p62 level was reduced by 60% with Rapa, an initiator of autophagy, treatment alone at 0.25 μM and was increased by 75% with CQ treatment at 40 μM (Fig. 6f). The mRFP-GFP-LC3 vector transfect assay combined with Hoechst 33258 staining results revealed that NBT induced apoptosis and elicited the complete autophagic process simultaneously in MCF-7 cells (Fig. 5c). In addition, we treated MCF-7 cells with 3-MA to inhibit the formation of autophagosomes at an early stage and CQ to inhibit the fusion of autophagy with lysosome at a later stage. Then, we elucidated the molecular mechanism of autophagy and mitochondrial apoptosis induced by NBT. The data showed that cotreatment with NBT and CQ significantly increased the apoptosis rate, Bax/Bcl-2 ratio, and the levels of Cyt C, cleaved-caspase-9, and cleaved-PARP compared with NBT alone. However, partially inhibiting autophagosome/mitophagosome formation with 3-MA significantly attenuated the NBT-induced caspase-9 and PARP activation in MCF-7 cells. These results indicated that NBT plus 3-MA inhibited the NBT-induced apoptosis, whereas NBT plus CQ could increase the apoptosis. The opposing effects of 3-MA and CQ on NBT-induced apoptosis might be because 3-MA can inhibit the formation of autophagosomes, whereas CQ can inhibit the fusion of autophagy with lysosome, resulting in the accumulation of autophagosomes. Therefore, we believed that NBT induced more apoptotic MCF-7 cells upon coincubation with the late-stage autophagy inhibitor CQ. Mitochondrial damage can induce the loss of mitochondrial membrane potential, resulting in the promotion of mitochondrial fission^54^. The dysregulation of mitochondrial fission and fusion has been reported to participate in the overall cellular viability and maintenance of mitochondrial homeostasis^55^. The expression of DRP1 and Fis1 was increased by CQ, whereas the Mfn2 level was decreased, which indicated that cotreatment with NBT and CQ could induce more mitochondrial fission in MCF-7 cells. To further investigate the mechanism of the effect of NBT and CQ combination on mitochondrial apoptosis, the effects on autophagy were tested. The results indicated that NBT induced the formation of autophagosomes, which promoted mitochondrial fission resulting in more apoptotic MCF-7 cells. Collectively, these results indicated that inhibiting the degradation of autolysosome by CQ potently increased the apoptotic activity of NBT via promoting mitochondrial fission induced by the excessive accumulation of mitophagosomes.

NBT could inhibit tumor growth in a mouse xenograft model in vivo. The maximum dosage of NBT was calculated to be 1875 mg/kg after no treatment-related toxic manifestations and mortality were observed in nude mice given NBT (i.p.) three times within 24 h. Then, after 11 days of NBT and BT administration, the results showed that NBT or BT was safe. The overall toxicological effects of NBT were not examined in this study but will be examined in our future study. The pharmacodynamical results indicated that NBT demonstrated higher antitumor potential than BT in vivo. In addition, hematoxylin and eosin (H&E) staining and TUNEL assay showed that NBT induced more necrotic and apoptotic cells than BT. Therefore, NBT was useful and safe in treating breast cancer, specifically by targeting the mitochondrial apoptosis pathway.

**Fig. 8:**
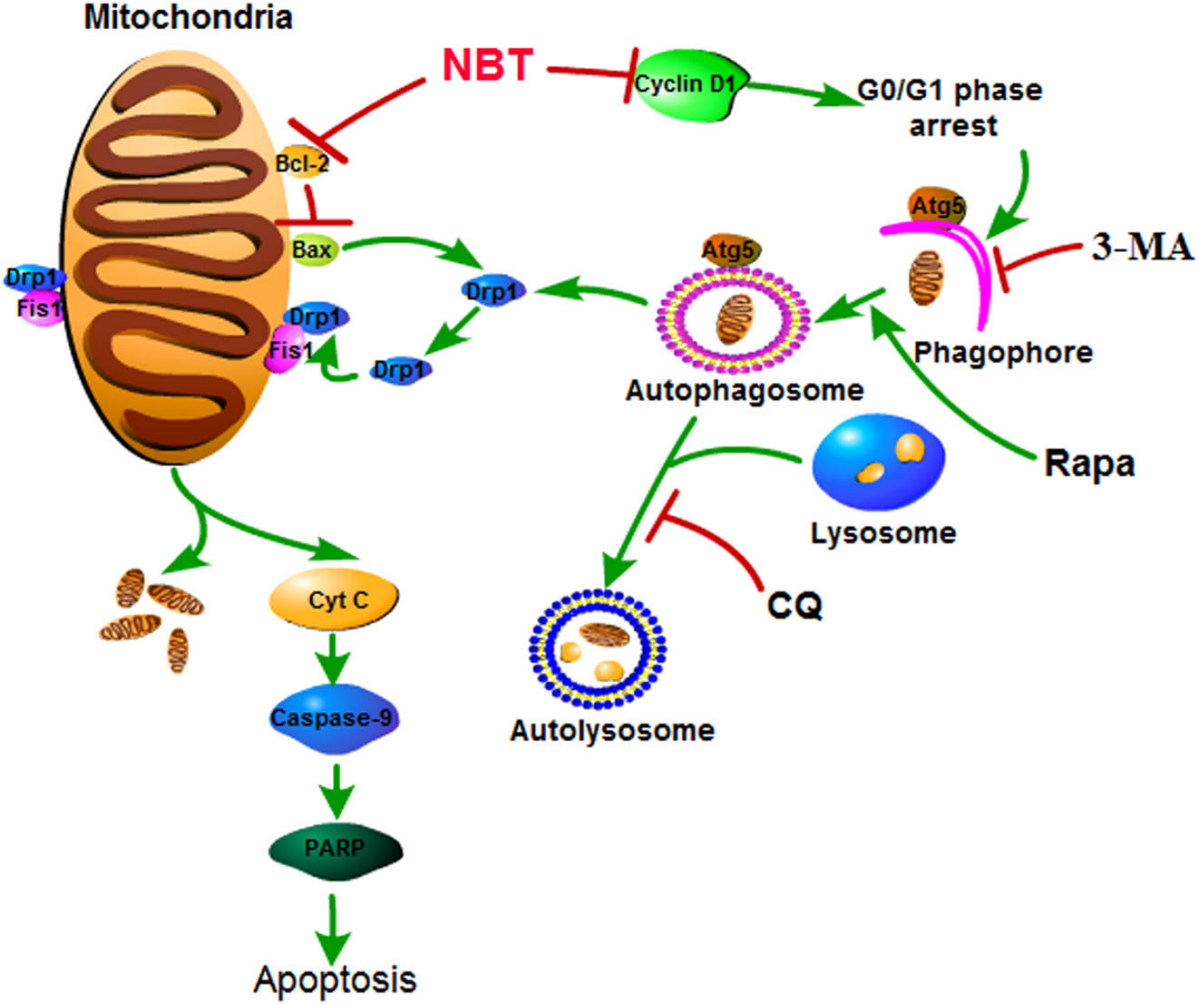
Proposed mechanisms for NBT-induced apoptosis and the synergistic effects of NBT and CQ agents

In summary (Fig. 8), cotreatment with NBT and CQ could significantly enhance the levels of Atg5, LC3 II, and p62; the number of mitochondria and mitophagosome; and the levels of DRP1, Fis1, and Cyt C compared with NBT or CQ treatment alone in MCF-7 cells. That is to say, the overexpression of Atg5 could increase the apoptosis induced by NBT by accelerating mitochondrial fission and inducing Cyt C release in MCF-7 cells. These findings suggest that NBT possesses antitumor potential for breast cancer via the mitochondrial pathway, and CQ accelerates NBT-induced apoptosis by promoting excessive mitochondrial fission. Our findings demonstrate that NBT is an excellent anticancer therapeutic candidate, and cotreatment with NBT and CQ is an effective therapy for breast cancer treatment. However, our study has some limitations, and more research will be carried out in the future.

## Materials and methods

### Reagents

Cell Counting Kit-8 (CCK-8) was from Dojindo (Kumamoto, Kyushu Island, Japan). The Annexin V-FITC Apoptosis Detection Kit, Cell Cycle Detection Kit (R.T.U), and Caspase-9 Colorimetric Assay Kit were purchased from KeyGen Biotech (Nanjing, China). Dimethyl sulfoxide (DMSO), Hoechst 33258, 3-MA, and CQ diphosphate salt were from Sigma-Aldrich (St. Louis, MO, USA). The cell lysis buffer for Western blot and IP, Prestained Dual Color Protein Molecular Weight Marker, and BeyoECL Plus were from Beyotime (Shanghai, China). The anti-cyclin D1 (ab134175, 1:4000 dissolution), anti-Bcl-2 (ab182858, 1:4000 dissolution), anti-Bax (ab32503, 1:5000 dissolution), anti-cytochrome (ab133504, 1:5000 dissolution), anti-LC3B (ab192890, 1:2000 dissolution), anti-DRP1 (ab184247, 1:1000 dissolution), anti-mitofusin 2 (ab124773, 1:5000 dissolution), anti-TTC11 (ab71498, 1:400 dissolution), and COX4I1 (ab16056, 1:1000 dissolution) were from Abcam Technology (London, England). Anti-Atg5 (#12994, 1:1000 dissolution), anti-p62 (#8025, 1:1000 dissolution), anti-caspase-9 (#9502, 1:1000 dissolution), anti-PARP (#9532, 1:1000 dissolution), and anti-rabbit IgG, HRP-linked antibody (#7074, 1:3000 dissolution) were from Cell Signaling Technology (Boston, MA, USA).

### Cell lines, growth conditions, and treatment

MCF-7, HeLa, A549, BEAS-2B, MCF10A, and L-02 cell lines were purchased from the American Type Culture Collection (ATCC) (Rockville, Maryland, USA). Cells were maintained in RPMI Medium 1640 basic/DMEM/high-glucose DMEM containing 10% fetal bovine serum, penicillin, and streptomycin (Gibco BRL, UK) in a CO_2_ humidified incubator (Sanyo, Japan) at 37 °C with 5% CO_2_. BT (purity >95%, BET201211233, Xiao Gan Shen Yuan ChemPharm Co., Ltd., Hubei, China) and NBT were dissolved in DMSO stored at 4 °C until use (DMSO, <0.1%).

### Cell proliferation testing

Cells were grown in 96-well plates at a density of 5 × 10^3^ cells/100 μL for 24 h, and then various concentrations of BT or NBT were added for 24 or 48 h. The growth inhibition of BT and NBT was tested with 10 μL CCK-8 per well. Optical density was measured at 450 nm by using a multimode plate reader (PerkinElmer EnSpire, USA).

### Flow cytometric analysis of cell cycle phase distribution

MCF-7 cells were seeded in six-well plates at a density of 2 × 10^5^ cells/2 mL for 24 h and exposed to 6, 12, and 24 μM concentrations of NBT. After 24 h, the cells were washed once with PBS (Gibco BRL, UK), collected using 0.25% trypsin/0.02% EDTA (Gibco BRL, UK), and centrifuged at 1500 r.p.m. for 5 min. Approximately 10^6^ cells were fixed in 70% cold ethanol at 4 °C overnight. Ethanol-fixed cells were again washed with PBS and resuspended in 100 μL RNAse A in a 37 °C water bath for 30 min and then were incubated with 400 μL PI at 4 °C for 30 min blocking the light ray and analyzed by using FACS (BD Bioscience, USA).

### Quantitative real-time PCR

MCF-7 cells were harvested after treatment with 6, 12, and 24 μM concentration of NBT for 24 h. Total RNAs were extracted by using TriZol (Takara Biotechnology, Japan). Next, 1 μg total RNA samples was reverse transcribed into cDNA by using PrimeScript™ RT Master Mix (Takara Biotechnology, Japan) and amplified by using SYBR Premix Ex Taq™ II (Takara Biotechnology, Japan) of cyclin D1 and GAPDH.

Cyclin D1, forward: 5′-CGTGTAGCTATGGAAGTTGCA-3′,

reverse: 5′-CCCGAATGAGAGTCCTACAG-3′;

GAPDH, forward: 5′-CGG AGTCAACGGATTTGGTCGTAT-3′,

reverse: 5′-AGCCTTCTCCATGGTGGTGAAGAC-3′ (Takara Biotechnology, Japan). RT-PCR was conducted under the following conditions: 95 °C for 5 s, annealing for 30 s at 60 °C, and cycling for 40 times. The RT-PCR results were normalized with GAPDH using ΔΔCT analysis.

### Hoechst 33258 staining

MCF-7 cells were seeded in a coverslip, treated, fixed in 4% paraformaldehyde at 4 °C for 20 min, washed twice with PBS, and then incubated with Hoechst 33258 fluorescent dye (10 μg/mL) at room temperature for 10 min. Images were taken with a fluorescence microscope (Leica, Germany).

### Flow cytometric analysis of apoptosis by annexin V with PI staining

MCF-7 cells treated with NBT for 24 h were collected by using 0.25% trypsin and washed twice with cold PBS (2000 rpm, 5 min). Approximately 1–5 × 10^5^ cells were resuspended in 500 μL binding buffer and then incubated with 5 μL annexin V-FITC with 5 μL PI at room temperature for 5–15 min. The samples were analyzed by flow cytometry using the FL1 (FITC) and FL3 (PI) laser lines.

### Mitochondrial and cytosolic fractionation

MCF-7 cells treated with NBT and/or CQ for 24 h were harvested, washed twice with cold PBS, and then fractionated into cytosolic and mitochondrial fractions by using a Qproteome Mitochondria Isolation Kit (Qiagen, Germany) following the manufacturer’s protocol.

### Protein measurement

MCF-7 cells treated with NBT and/or CQ for 24 h were lysed in cell lysis buffer containing 1% PMFS for 30 min on ice and centrifuged at 12,000 r.p.m. for 5 min at 4 °C. Then, the protein was quantified by using a BCA Protein Assay Kit (BestBio, Shanghai, China).

### Caspase-9 activity assay

Caspase-9 activity assay was conducted by using a caspase-9 colorimetric assay kit. Approximately 3–5 × 10^6^ MCF-7 cells treated with NBT for 24 h were harvested, washed twice with PBS at 2000 r.p.m. for 5 min, lysed in cold lysis buffer on ice for 20–60 min, and centrifuged at 10,000 r.p.m. for 1 min at 4 °C. A total of 100 μg of protein (BCA method) were mixed with 50 μL 2× reaction buffer containing 0.5 μL DTT and 5 μL caspase-9 substrate. The mixture was incubated at 37 °C in the dark and then measured with a multimode plate reader at 405 nm.

### Western blots

Total cell extracts (50 μg) were separated by using SDS-PAGE at 120 V for 1.5 h at room temperature and then eletrotransferred onto PVDF membranes at 300 mA for 1 h at 4 °C. The membranes were washed three times with TBST each for 10 min, blocked with 5% nonfat dry milk in TBST for 2 h at room temperature, rinsed with TBST, incubated with primary antibodies overnight at 4 °C, washed three times with TBST, incubated with HRP-linked anti-IgG for 1 h at room temperature, and then washed three times as before. At the same time, β-actin, GAPDH, and tubulin acted as the control. Afterward, the chemiluminescent bands were analyzed using Image Lab™ Software on ChemiDoc XRS + (Bio-Rad, USA).

### Flow cytometric measurement of mitochondrial membrane potential by JC-1 staining

5,5′,6,6′-Tetrachloro-1,1′,3,3′-tetraethylbenzimidazolylcarbocyanine odide (JC-1) retention in mitochondria is driven by ΔΨm, which determines cell population with integrated mitochondrial functions. However, JC-1 is released from mitochondria when its membrane potential depolarizes. Less than 1 × 10^6^ MCF-7 cells treated with NBT for 24 h were collected, washed twice with PBS at 2000 r.p.m. for 5 min, and incubated with JC-1 for 20 min in a CO_2_ incubator at 5% CO_2_/37 °C. Then, the cells were washed twice in 1× incubation buffer at room temperature (2000 r.p.m., 5 min) and resuspended in 500 μL 1× incubation buffer. The changes in mitochondrial membrane potential (Δψm) were measured with a flow cytometer in FITC and PI channels (EX = 488 nm, EM = 530 nm).

### TEM

Cells were collected with 1 mL serum-free medium and fixed in 2.5% glutaraldehyde at room temperature for 1 h and then 4 °C for 3 h, where glutaraldehyde was replaced with PBS. The samples were examined by using a TEM.

### DNA transfection

Transfection was achieved using Lipofectamine 2000 Transfection Reagent (Invitrogen, Carlsbad, CA, USA) according to the manufacturer’s protocol. A total of 1 × 10^4^ of MCF-7 cells cultured on coverslips were transduced with 0.8 μg of mRFP-GFP-LC3 plasmid for 48 h and then treated with NBT or NBT + CQ or Rapa or Rapa + CQ for another 24 h. Then, the cells were stained with Hoechst 33258 fluorescent dye. Images were taken by using a fluorescence microscope (Leica, Germany).

### In vivo antitumor efficacy of NBT on MCF-7 tumor-bearing nude mice

β-Estradiol (0.03 mg/0.2 mL PBS solution) was intraperitoneally injected to female BALB/c nude mice. After 5 days, MCF-7 cells (10 × 10^6^) were injected subcutaneously into the nude mice under the left axillae. The mice were randomly divided into four different groups (*n* = 8), namely, normal saline (NS) group (0.2 mL i.p.), 5-FU-positive control group (22 mg/kg i.p.), BT group (100 mg/kg i.p.), and NBT group (100 mg/kg i.p.), when the tumor size reached 80 mm^3^ after 5 days of inoculation of MCF-7 cells in nude mice. The mice were treated with NS, 5-Fu, BT, and NBT every day for 11 days. Tumors were measured every other day with the help of a vernier caliper (calculated volume (mm^3^) = Length (mm) × Width (mm)^2^/2). After the last treatment, the tumor of each mouse was isolated and fixed in 10% formalin.

### H&E staining

Tumor samples were fixed in 10% formalin, paraffinized, and sectioned into 4-μm sections, which were stained with H&E according to standard methods.

### TUNEL assay

Tumor samples were fixed in 10% formalin, paraffinized, and sectioned into 4-μm sections, which were stained with FITC-POD according to standard methods.
